# MicroRNAs and Cardiovascular Disease Risk

**DOI:** 10.1007/s11886-023-02014-1

**Published:** 2024-01-11

**Authors:** Charles D. Searles

**Affiliations:** Emory University School of Medicine and Atlanta VA Health Care System, 1670 Clairmont Road, Decatur, GA 30033 USA

**Keywords:** MicroRNA, Extracellular microRNA, Biomarker, Cardiovascular disease, Coronary artery disease, Heart failure, Arrhythmia

## Abstract

**Purpose of Review:**

MicroRNAs (miRNAs)—short, non-coding RNAs—play important roles in almost all aspects of cardiovascular biology, and changes in intracellular miRNA expression are indicative of cardiovascular disease development and progression. Extracellular miRNAs, which are easily measured in blood and can be reflective of changes in intracellular miRNA levels, have emerged as potential non-invasive biomarkers for disease. This review summarizes current knowledge regarding miRNAs as biomarkers for assessing cardiovascular disease risk and prognosis.

**Recent Findings:**

Numerous studies over the last 10–15 years have identified associations between extracellular miRNA profiles and cardiovascular disease, supporting the potential use of extracellular miRNAs as biomarkers for risk stratification. However, clinical application of extracellular miRNA profiles has been hampered by poor reproducibility and inter-study variability that is due largely to methodological differences between studies.

**Summary:**

While recent studies indicate that circulating extracellular miRNAs are promising biomarkers for cardiovascular disease, evidence for clinical implementation is lacking. This highlights the need for larger, well-designed studies that use standardized methods for sample preparation, miRNA isolation, quantification, and normalization.

## Introduction

Cardiovascular disease (CVD) encompasses a range of disorders that impact the heart and vasculature and is a leading cause of morbidity and morbidity worldwide. Identifying reliable biomarkers for assessing and managing CVD risk is crucial for early diagnosis and intervention. MicroRNAs (miRNAs) have emerged as pivotal regulators of gene expression and modulators of complex cellular processes in health and disease. MiRNAs are known to target and regulate expression of genes involved in pathophysiologic processes that contribute to the development of CVD, including inflammation, oxidative stress, lipid metabolism, and endothelial function. Dysregulation of miRNA expression has been linked to a range of cardiovascular diseases, including coronary artery disease (CAD), myocardial infarction, arrhythmia, and heart failure. The ability of miRNA expression to reflect changes in cellular processes, the tissue-specific expression of miRNAs, and the presence of miRNAs in blood and other body fluids that can be easily measured make miRNAs attractive candidates as biomarkers and therapeutic targets for CVD.

This review highlights studies underlying the current state of knowledge regarding the role of miRNAs in CVD pathophysiology and the potential of miRNAs as biomarkers for assessing CVD risk. In addition, the challenges and opportunities in harnessing extracellular miRNAs for diagnostic and prognostic applications in the context of CVD are discussed.

## miRNA Biogenesis and Function

MiRNAs are short (~ 19–23 nucleotides), non-coding RNAs that modulate the expression of target genes through repressing translation or inducing degradation of specific messenger RNAs (mRNAs) [1–3]. MiRNAs were discovered by Ambros and colleagues in 1993 when they showed that a gene, named *lin-4*, involved in the development of *Caenorhabditis elegans* (*C. elegans*) worm was actually a small, non-protein coding RNA molecule [4]. Since the discovery of this miRNA, miRNAs have been identified across every plant and animal species and have been shown to be highly conserved, reflecting their importance as regulatory molecules. MiRNAs are now recognized to modulate most aspects of cellular homeostasis and physiology, including differentiation, growth, proliferation, and apoptosis [5], and over 60% of coding genes in humans are computationally predicted to be targeted by miRNAs [6]. MiRNAs are regarded as fine-tuners of genes involved in just about every physiologic and pathophysiologic process [7].

The biogenesis of miRNAs begins in the nucleus where primary miRNA transcripts (pri-miRNAs) are generated from various genomic loci [8] by RNA polymerase II and III [9]. Subsequently, the microprocessor complex, consisting of the RNase III enzyme, Drosha, and its cofactor DGCR8 (DiGeorge Syndrome Critical Region 8), cleaves the pri-miRNA. The resulting product is a pre-miRNA, which varies in length between 60 and 70 nucleotides. The pre-miRNA has a stem-loop hairpin structure that consists of a double stranded stem (approximately 33 nucleotides) and a single stranded loop [10]. Pre-miRNA is exported from the nucleus to the cytoplasm by Exportin-5-Ran-GTP where the RNase III endonuclease Dicer cleaves the pre-miRNA terminal loop to produce a transient double stranded miRNA that is loaded on the RNA-induced silencing complex (RISC) [11]. Argonaute protein separates the two complimentary mature miRNAs (miRNA-5p and miRNA-3p) into guide and passenger strands [12]. The RISC leads the guide strand to specific mRNA targets through base pairing of the miRNA seed sequence, defined as nucleotides 2–8 of the 5′ end, with the complementary sequence in the target mRNA, typically located in the 3′ untranslated region. This interaction can either inhibit target mRNA translation or induce its degradation [13–15]. Since miRNA-mediated gene suppression typically requires only seven to eight nucleotides, a single miRNA can regulate the expression of hundreds of mRNAs [16], and many different miRNAs can regulate a single mRNA [6]. Thus, miRNA-mediated gene repression is complex and can involve regulation of entire gene networks.

While most miRNAs are intracellular, miRNAs can be found extracellularly in plasma and other body fluids [17–20], a finding that has generated intense interest in extracellular miRNAs as biomarkers for a number of diseases, including cancer, myocardial infarction, heart failure, and Alzheimer’s disease [21–27]. However, extracellular miRNAs are also likely to play important roles as paracrine or endocrine signals during the disease process because they are capable of being transferred to recipient cells with a subsequent change in gene expression and cellular function [28–34, 35••]. Extracellular miRNAs are released from cells in a tightly regulated manner [33, 36–38], with only select intracellular miRNAs exported in response to a specific biological stimuli [31, 33, 37, 39]. This concept is supported by studies showing a detectable shift in circulating miRNA profiles in various diseases, including CAD [38, 40–43].

Extracellular miRNAs in plasma are released from multiple cell types, including platelets, erythrocytes, immune cells, myocytes, and endothelial cells, and are protected from degradation by encapsulation in cell-derived extracellular vesicles (EVs) [31, 33, 44] or by forming complexes with circulating proteins or lipoproteins [32, 45]. Extracellular miRNAs are remarkably stable, even if the sample has been left at room temperature for a prolonged period of time or has been exposed to multiple freeze/thaw cycles [19, 46]. Certain extracellular miRNA transport modalities may be more likely to participate in cell-to-cell signaling [44, 47], whereas others are likely reflective of tissue injury and cell death [40]. Platelets, RBCs, immune cells, and ECs release different subpopulations of EVs into the circulation in response to cellular activation or apoptosis [48]. Microvesicles are EVs that are generated by plasma membrane blebbing and have specific markers of the cell of origin. Exosomes are EVs released from the fusion of multivesicular bodies with the plasma membrane, and apoptotic bodies are EVs generated during the final stages of programmed cell death. However, because no straightforward criteria exist to distinguish, isolate, and identify subpopulations of cell-derived vesicles, the term extracellular vesicle is used to collectively refer to cell-derived vesicles present in body fluids [49].

## MiRNAs in Cardiovascular Pathophysiology

MiRNAs play multifaceted roles in the development and progression of CVD. Dysregulated miRNAs contribute to endothelial dysfunction, vascular inflammation, oxidative stress, and fibrosis—key drivers of atherosclerosis and other CVD pathologies. MiRNAs also participate in cardiac remodeling processes, influencing cardiomyocyte hypertrophy, fibroblast activation, and extracellular matrix deposition.

In endothelial cells (ECs) and vascular smooth muscle cells (VSMCs), miRNA expression is regulated by various pro- and anti-atherogenic stimuli [50–55]. Functional studies indicate that miRNAs regulate pathological processes in these cells that contribute to atherosclerosis [56], including apoptosis, senescence, angiogenesis, arterial remodeling, and vascular inflammation [57, 58]. Furthermore, miRNA signatures have been shown to differ between stable and unstable atherosclerotic plaques [59], suggesting that miRNA modulation may be an important therapeutic target for atherosclerosis.

In ECs, the expression of several miRNAs has been shown to be sensitive to mechanical forces. These mechanical forces, such as shear stress, are typically associated with steady, unidirectional blood flow, which is anti-inflammatory, or low, disturbed blood flow, which is pro-inflammatory. An example of a mechano-sensitive miRNA that is highly expressed in ECs is miR-92a, a member of the miR-17 ~ 92 cluster. Expression of miR-92a was increased in ECs exposed to pro-inflammatory shear stress forces and oxidized LDL, which led to suppression of the transcription factors KLF2 and KLF4. Inhibition of miR-92a expression reduced inflammation in cultured ECs and atherosclerotic mice [60]. MiR-126 is another flow-dependent miRNA that is abundantly expressed in ECs and has been implicated in modulating genes involved in vascular inflammation and angiogenesis. Systemic delivery of miR-126 to hyperlipidemic mice promoted endothelial proliferation and inhibited atherosclerotic lesion progression [61]. Other mechano-sensitive miRNAs implicated in modulating endothelial inflammation and atherosclerosis progression include miRs-712/-205, -10a, -663, and -155 [50, 54, 62–65].

Endothelial expression of adhesion molecules, which facilitate leukocyte recruitment to the blood vessel wall, is an early hallmark of atherosclerosis, and at least two miRNAs (miRs-17 and -31) have been shown to directly target adhesion molecules in ECs. However, the role of these miRNAs in experimental models of atherosclerosis is unknown [66]. In contrast, two other miRNAs, miRs-181b and -146a, that each modulate the pro-inflammatory NF-kappaB signaling pathway in ECs, have been demonstrated to suppress atherosclerosis in animal models [67, 68].

In VSMCs, miRs-143 and -145 are co-transcribed as single pri-miRNA transcript and are among the highest expressed miRNAs in this cell type. These miRNAs are considered major regulators of VSMC contractile function [69], and overexpression of miR-145 in hyperlipidemic mice reduced atherosclerotic plaque formation [70]. In contrast, miRs-221 and -222 have been shown to promote VSMC proliferation and mice deficient in these miRNAs had reduced VSMC proliferation and neointimal lesion formation after mechanical injury [71]. Similarly, pharmacologic inhibition of miR-21 reduced neointimal lesion formation in response to mechanical injury in mouse models [72].

Imbalances in cholesterol homeostasis can promote accumulation of cellular cholesterol and lead to the development of atherosclerosis. Several liver-enriched miRNAs have been identified that can modulate lipoprotein metabolism and alter the development of atherosclerosis in animal models. These miRNAs include miRs-122, -223, and -27b, all of which have an impact on cholesterol biosynthesis [73–76]. MiR-30c, which modulates apoB-containing lipoproteins and can decrease levels of plasma total and LDL cholesterol, was also shown to reduce atherosclerosis in hyperlipidemic mice [77]. Other liver-enriched miRNAs have been linked to the development of atherosclerosis through regulation of the LDL receptor (miR-148a, miR-128–1) [78, 79] and HDL levels (miR-33) [80]. Together, these studies highlight the different roles of miRNAs in cholesterol homeostasis and the potential utility of therapeutically targeting these miRNAs for management of dyslipidemias and atherosclerosis.

Another group of miRNAs have been identified that are predominantly expressed in myocytes and have an important role in the development and function of cardiac muscle tissue. These miRNAs, which include miRs-1, -133a, -133b, -206, -208a, -208b, -499a, and -499b, are known as myomiRs and have been linked to CVD. MiR-1, which accounts for approximately 40% of heart miRNAs [81], and miR-133 have been implicated in heart development, but dysregulated expression of these miRNAs has been found in cardiac hypertrophy, myocardial infarction, and arrhythmias [82]. miRs-208a/208b and miR-499 are miRNAs encoded within myosin heavy chain genes, MYH6 and MYH7, respectively, and have roles in cardiac stress response and differentiation. MiR-499 targets SOX6, which has a significant role in cardiomyocyte viability, proliferation, and apoptosis [83]. Altered expression of miR-208 has been linked to cardiac fibrosis, hypertrophy, arrhythmia, myocardial infarction, and heart failure [84]. Cardiac overexpression of miR-195 was shown to promote dilated cardiomyopathy [85]. Other miRNAs involved in the development of myocardial fibrosis include miRs-21, -135b, and -29 [86]. Interestingly, miR-29 has also been shown modulate the development of atrial fibrillation [87]. Overall, the studies described here and others have shown that miRNA expression profiles in cardiac tissues are altered in the development of heart failure and arrhythmia, reflecting the involvement of these miRNAs in regulating signaling pathways that control cardiac remodeling, contractility, and electrical signaling.

## Extracellular miRNAs as Biomarkers

Over the last 10 to 15 years, numerous studies have investigated the utility of extracellular miRNAs in blood as non-invasive biomarkers for CVD diagnosis and prognosis. The focus on circulating miRNAs has been due to several features of extracellular miRNAs, including high stability, resistance to degradative enzymes, ease of detectability, and expression levels in plasma or serum that likely reflect CVD-related changes in intracellular miRNA expression. Repositories of human tissues and biofluids have been essential resources for work on miRNA biomarker discovery, and archived plasma or serum has been utilized in many extracellular miRNA studies [88–90].

### Acute MI and ACS

Among the earliest studies investigating extracellular miRNAs as diagnostic biomarkers for cardiovascular disease were those involving participants with acute myocardial infarction (AMI) and acute coronary syndrome (ACS). In 2010, Ai and coworkers reported that miR-1 was elevated in the plasma of participants with AMI [91]. Subsequently, additional studies, focusing on myomiRs (miRs-1, -133a, -208a, -208b, -499), examined the clinical usefulness of elevations in extracellular miRNAs as early biomarkers of AMI and ACS [40]. In AMI, levels of myomiRs (e.g., miR-208b), which should be low or undetectable in the absence of myocardial injury, were elevated in plasma by 3 h after symptoms and could remain elevated over 90 days [22]. Wang et al. reported that miRs-1, -133, -499, and -208a were elevated in the plasma of patients with AMI but were no better than troponin in diagnosing AMI [92]. Other studies, comparing troponin to one or a combination of these miRNAs in diagnosing AMI and ACS, have had variable results, with some studies showing better test statistics for extracellular miRNAs compared to troponin [93, 94], while others showing worse performance of extracellular miRNAs [95, 96]. Overall, it seems that, at best, the performance of extracellular miRNAs as biomarkers for AMI and ACS is no better than troponins. While extracellular miRNAs might not improve diagnostic accuracy of AMI compared to troponin, there are some promising data that indicate circulating levels of miRNAs associated with atherosclerosis might help discriminate MI subtype (i.e., type I vs type II). For example, total blood levels of miR-663b, an EC-enriched, mechano-sensitive miRNA implicated in atherosclerosis, demonstrated 95% sensitivity, 90% sensitivity, and 90% accuracy in differentiating AMI participants from controls [97]. These data suggest that non-cardiomyocyte specific miRNAs linked to atherosclerosis may help in discriminating MI subtype, but much more work needs to be done in this area.

### Chronic Coronary Artery Disease

There has been considerable interest in the ability of circulating miRNAs to predict the presence coronary artery disease (CAD) and incident myocardial infarction (MI). In 2010, Fichtlscherer and coworkers compared the plasma miRNA profile of eight participants with stable CAD to that of eight healthy controls. In the CAD group, levels of miRs-126, -17, -92 (EC-enriched miRNAs), -145 (VSMC-enriched miRNA), and -155 were all reduced compared to those in control participants, while levels of miRs-133 and -208a (cardiomyocyte-enriched miRNAs) were increased compared to controls [23]. Other groups have reported reduced expression of miRNAs linked to atherosclerosis in whole blood of patients with CAD compared to healthy subjects [43]. Similarly, Zhu et al. found that levels of miR-155 in plasma or peripheral blood mononuclear cells (PBMCs) were lower in patients with unstable angina or AMI compared to those in patients with chest pain syndrome; miR-155 levels were further reduced in patients with two or three vessel disease compared to patients with zero to one vessel disease [98]. In contrast, another group reported that patients with stable or unstable angina had increased levels of plasma miRNAs (miRs-1, -122, -126, -133a, -133b, -199a, -337, -433, and -485) compared to controls. Different combinations of this panel could correctly classify subjects with stable angina compared to controls and subjects with unstable angina compared to controls in greater than 87% of cases [99], but no combination could discriminate unstable angina from stable angina. While these studies suggest that circulating miRNA profiles have the potential to discriminate patients with angiographically documented CAD from those without significant CAD, there is considerable variability in the miRNA profiles reported. Currently there is no consensus on which miRNAs are best indicators of CAD, and additional studies are required before circulating miRNA profiles can be used for this purpose clinically.

Several studies have examined the association between basal circulating miRNA levels and incident MI. Zampetaki et al. reported a significant relationship between plasma levels of miRs-126, -197, and -223 and MI in participants of the Bruneck Study, which had a 10-year follow-up period. Interestingly, this group determined that the source of these plasma miRNA was platelets [89]. Bye and coworkers assessed the utility of serum miRNA levels in predicting fatal AMI within the HUNT study cohort, which had a 10-year observation period [100]. Using real time polymerase reaction (RT-qPCR), this group identified 10 miRNAs that were differentially expressed between participants that had fatal MI and risk factor matched controls who did not. Two of these extracellular miRNAs (miRs-424 and -26a) had a sex-specific association with risk of fatal MI, and a model consisting of five miRNAs with highest ability to predict future AMI in both sexes was identified (77.6% correct classification). Adding these five miRNAs to the Framingham Risk Score (FRS) significantly increased the receiver operating characteristic (ROC) area under the curve (AUC). Subsequently, this group identified another five serum miRNAs (miRs-21, -26a, -29c, -144, and -151a) that, along with FRS, could predict fatal or non-fatal MI (ROC AUC 0.68) [101].

Keller et al. assessed the ability of a different panel of five extracellular miRNAs (miRs-34a, -223, -378, -499, and -133) to predict overall mortality and/or cardiovascular events. All of the miRNAs in this panel were known to be associated with different CVD pathophysiology, and the investigators used RT-qPCR to study plasma from participants of the DETECT and SHIP studies [102]. A score based on the miRNA panel showed association with overall mortality, independently of established risk scores, such as FRS or SCORE. Adding the panel of five miRNAs to risk stratification models based on FRS or SCORE was able to improve prediction of mortality. Another group tried to identify plasma miRNA signatures associated with major adverse cardiovascular events (MACE) in 60-year-old participants of the Stockholm Study [103]. This group identified 16 interacting miRNA pairs associated with MACE; miR-320b was present in all interacting pairs, with increasing levels of this miRNA associated with progressive increase in MI risk. Wang and coworkers screened pooled samples of serum or PBMCs from 10 CAD patients and 10 healthy controls, and ECs exposed to hypoxia [104]. The authors found five miRNAs (miRs-10a, -126, -210, -423, and -92a) that were detected in serum and dysregulated in PBMCs of CAD patients and hypoxic ECs. They tested these five miRNAs in separate cohorts of CAD patients and controls and identified miRs-10a and -423 as best candidates for predicting CAD, but only miR-423 was associated with risk of non-fatal MI. Combining miR-423 with traditional risk factor-based models improved prediction performance.

### Heart Failure

A potential role for extracellular miRNA in the diagnosis and prognosis of heart failure has been reported in a number of studies. An early report by Tijsen and coworkers studied plasma extracellular miRNA profiles in patients with heart failure compared to those in patients with dyspnea not related to heart failure and healthy controls. They found that elevated levels of miR-423 was a diagnostic predictor of heart failure [27]. Subsequently, other groups have reported the diagnostic utility of measuring other extracellular miRNAs in heart failure, including miRs-1, -21, -126, and -499 [105–107]. Two groups have examined plasma miRNA profiles in participants with heart failure preserved ejection fraction (HFpEF) versus those with heart failure reduced ejection fraction (HFrEF). Wong et al. identified six miRNAs that discriminated HFpEF from controls and four miRNAs (miRs-125a, -190a, -550a, and -638) that distinguished HFpEF from HFrEF [108]. The selective miRNA panels showed stronger discriminative power than N-terminal pro-brain natriuretic peptide. Watson et al. identified five miRNAs (miRs-375, -146a, -30c, -328, and -221) that were differentially expressed between HFpEF and HFrEF. None of these miRNAs outperformed brain natriuretic peptide in predicting heart failure, but the discriminative value of brain natriuretic peptide was improved by use in combination with any of the miRNAs alone or in a panel.

### Arrhythmia

As discussed above, intracellular miRNA expression has been shown to be important in the development of arrhythmia, including miR-328, which was found to be elevated in the atrial myocardium of a canine model of atrial fibrillation [109]. MiR-328 was also elevated in the right atrium of patients with atrial fibrillation undergoing open-heart surgery. Overexpression of miR-328 increased vulnerability to atrial fibrillation through regulation of genes encoding L-type calcium channels [109]. Subsequently, several groups have also examined whether circulating miRNAs can serve as biomarkers for atrial fibrillation. McManus and coworkers examined whole blood miRNA profiles in participants of the Framingham Heart Study and found that levels of miRs-328, -150, -331, and -28 were lower among participants with prevalent atrial fibrillation, but only lower miR-328 levels were significant after adjustments for age, sex, and technical factors. Liu et al. examined the plasma miRNA expression profiles in patients with atrial fibrillation and found that only miR-150 was significantly lower in these patients compared to healthy controls. Other groups have identified extracellular levels of a variety of miRNAs, including miRs-214, -342 [110], -29b, -21 [87], and miR-150 [111], as predictors of atrial fibrillation. While there was little overlap between these studies in extracellular miRNAs predictive of atrial fibrillation, it should be noted that the clinical profile of the cohorts studied were also considerably different. One final biomarker study worth mentioning is that of Silverman and coworkers, who examined the plasma extracellular miRNA profiles associated with sudden cardiac and/or arrhythmic death [112•]. In a nested case control study of patients with coronary artery disease and followed prospectively for sudden cardiac death (SCD), the authors found that levels of miRs-150, -29a, and -30a were associated with a 4.8-fold increased risk of SCD. Interestingly, their bioinformatics analysis linked these miRNAs to apoptosis, fibrosis, and inflammation, thus supporting the biological basis of these biomarkers.

## Challenges of Extracellular miRNAs as Biomarkers for CVD Risk and Prognosis

While the studies of human blood discussed above indicate that extracellular miRNAs are promising non-invasive biomarkers for CVD risk and prognosis, the extracellular miRNA profiles identified thus far have generally shown poor reproducibility between studies. These inconsistencies hinder clinical application of extracellular miRNA profiles and are likely largely due to methodological variations in measurement of extracellular miRNAs. The development of extracellular miRNAs as clinical biomarkers will be critically dependent on identifying factors responsible for inter-study variability and establishing standardized protocols for measurement of extracellular miRNAs.

Sample processing has a significant influence on miRNA levels detected in plasma or serum. Cheng et al. demonstrated that the protocol for preparing plasma can dramatically influence levels of miRNA detected in plasma by affecting the number of residual platelets in the sample [113]. Platelets are a rich source of miRNA, and most miRNAs detected extracellularly in plasma are also expressed in platelets. Furthermore, miRNA levels in platelets are often markedly much higher than the extracellular levels [113, 114], so release of intracellular miRNAs from residual platelets will have a major effect on extracellular miRNA levels. Our group demonstrated that freezing plasma samples containing residual platelets resulted in irreversible contamination of extracellular miRNA with intracellular platelet miRNA, and, consequently, miRNA levels in improperly processed plasma samples could largely be a reflection of the baseline platelet count in whole blood and not disease-induced changes in miRNA expression [115]. Current recommendations for extracellular miRNA profiling call for platelet poor plasma (PPP), generated by centrifuging whole blood (collected in EDTA or sodium citrate vacutainers) twice prior to freezing—the first centrifugation removes the bulk of circulating cells and the second removes residual platelets. Therefore, a critical factor for inter-study variability in extracellular miRNA profiles is likely sample preparation. For many of these studies, details of sample preparation and storage are not clear and residual platelet removal was either not performed or not reported [108, 112•, 115]. Slight differences in sample processing, such as centrifugation speed and sample volume, could result in systematic differences in the number of residual platelets and lead to batch effects. This is of particular concern in studies that used archived samples prepared at different sites, each using different sample preparation protocols and equipment.

The choice of anticoagulant and blood fraction can also affect quality of results in extracellular miRNA profiling studies. One study comparing miRNA levels between plasma and serum indicated that profiles in the two fractions may be largely similar, although some differences do exist [116]. A concern with using serum for biomarker studies is that platelet activation during clot formation can lead to the release of platelet miRNA into the serum. As far as anticoagulant for profiling of extracellular miRNA in plasma, either EDTA or sodium citrate are recommended, but sodium citrate may be preferred because there is concern that EDTA can cause artifactual release of miRNA from red blood cells and platelets [115, 117].

Another important factor affecting reproducibility of extracellular miRNA profiling studies is the strategy for normalizing miRNA expression in the sample, which tends to vary between studies. Intracellular miRNA expression in cells or tissues are typically normalized to expression of endogenous small RNA, such as small nucleolar RNA U6. However, currently there is no established endogenous extracellular small RNA or miRNA control, despite several studies that have used this strategy for normalization [118]. Other studies have normalized extracellular miRNA expression to total RNA concentration in the sample, as assessed by spectrophotometry [119, 120]. However, this approach can be unreliable because the amount of RNA extracted from plasma samples is usually well below the limits of accurate spectrophotometric quantification [121–123]. Extracellular miRNA levels have also been normalized to exogenous, non-human miRNA that has been spiked into the sample during RNA isolation, but this strategy may be unreliable when quantification of the extracted RNA is not possible. Lastly, when high-throughput data is available, such as data from RNA sequencing or miRNA arrays, global mean/median normalization methods have been used [124, 125], but this strategy produces results that are difficult to compare to other normalization methods. Clearly, these different normalization methods, all of which have been used in the studies discussed above, can profoundly affect extracellular miRNA profiles.

A final consideration for inter-study variability and why it has been difficult to achieve consensus on extracellular miRNAs profiles predictive of CVD is that the clinical outcomes in the different studies have often been different, ranging from cardiac events to fatal or non-fatal MI to all-cause death. Also, there has been considerable inter-study variability in ethnic/racial/sex diversity of study cohorts, study design, detection methods, and statistical analysis. Furthermore, standard ranges for levels of different extracellular miRNAs in blood have not been established.

## Conclusions

MiRNAs represent a dynamic and versatile class of molecules with significant potential in shaping the landscape of CVD risk assessment and management. Since the discovery of miRNAs, a great deal has been learned about miRNA biogenesis, function, and the importance of miRNA expression in the physiologic and pathophysiologic processes underlying cardiovascular health and disease. This review has highlighted some of the studies that have contributed to this knowledge but the referenced studies are not exhaustive and the reader is encouraged view other recent reviews on this subject.

Based on our ever increasing understanding of the roles of miRNAs in CVD, the tissue specificity of miRNAs, and the ease of miRNA expression assessment in plasma and other body fluids, extracellular miRNAs hold promise as both diagnostic tools and therapeutic targets for managing cardiovascular disease. The review highlights studies of patients with ACS, AMI, CAD, heart failure, and arrhythmia that provide evidence to support this promise. However, currently, evidence for clinical implementation of extracellular miRNAs as biomarkers for CVD is lacking. Extracellular miRNAs profiles have not been shown to increase the diagnostic or prognostic power of established biomarkers, such as LDL cholesterol, high sensitivity troponins, and N-terminal pro-brain natriuretic peptide. Furthermore, the field suffers from inconsistent results and significant inter-study variability. While this may be due, in part, to the relatively low concentrations of extracellular miRNAs, the main issues facing this field is lack of standardization of methodologies for miRNA measurement and variability in clinical endpoints assessed. For extracellular miRNAs to reach the status of robust biomarkers for personalized cardiovascular care, these issues will need to be addressed through well-designed clinical studies in larger prospective cohorts of patients, using standardized protocols.
